# The transfer of ^241^Am and ^137^Cs to the tissues of broilers’ organs

**DOI:** 10.1371/journal.pone.0235109

**Published:** 2020-07-01

**Authors:** Ainur S. Mamyrbayeva, Zhanat A. Baigazinov, Sergey N. Lukashenko, Andrey V. Panitskiy, Seil S. Karatayev, Anton N. Shatrov, Symbat A. Baigazy, Assem B. Bazarbayeva, M. Hegedűs, E. Tóth-Bodrogi, T. Kovács

**Affiliations:** 1 Institute of Radiation Safety and Ecology NNC RK, Kurchatov, Kazakhstan; 2 Institute of Radiochemistry and Radioecology, University of Pannonia, Veszprém, Hungary; 3 Russian Institute of Radiology and Agroecology, Obninsk, Kaluga region, Russian Federation; University of South Carolina, UNITED STATES

## Abstract

Data on the transfer of artificial radionuclides from the environment to the food supply is necessary for internal dose assessment. There is a necessity for expanding and improving the available information on these factors in order to make better dose models for specific scenarios. This paper describes the results of a field experiment with broiler chickens on the transfer factor (F_*f*_) and concentration ratio (C_R_) for the long-term intake of ^241^Am and ^137^Cs with grass meal and soil. The broilers were divided into two groups, each group had nine subgroups and each subgroup had three broilers. The radionuclide concentrations in the feed and the thigh muscle, thigh bone, and liver of 54 broilers divided between the grass meal and soil groups were evaluated by gamma spectrometry for 241Am and 137Cs. The duration of feeding with “contaminated” sources ranged between 1–70 days. The equilibrium stage of ^241^Am in muscle and bone occurs on the 1^st^ and 40^th^ day, respectively; for ^137^Cs in muscle– 30^th^ days of intake and for liver and bone– 7^th^ days. For ^241^Am, the liver did not reach equilibrium stage during the 70 days of intake. F_*f*_ of ^137^Cs in the “forage-muscle” and “soil-muscle” systems were determined as 1.9±0.3 and 0.18±0.05; F_*f*_ of ^241^Am in the “soil-muscle” system was—7.5×10^−5^.

## 1. Introduction

Farm animals’ products, such as meat, milk, eggs etc., produced on contaminated territory are one of the main sources of artificial radionuclide intake into a body of people [1, 2]. Therefore, migration parameters of radionuclides are still one of the main topics in the world community in case of radiological impact assessment. One gap in our knowledge on it is that there is still not enough data on the transfer processes for some specific situations [3], such as on the Semipalatinsk Test Site (STS), where the required data is on the transfer parameters of transuranic elements [4], for instance. This topic is complicated by the fact that some radionuclides have a big range of reported transfer factors into the same products; in some cases, the range could reach four orders of magnitude [5].

After analyzing the main publications on current world experiences [1–2, 5–7] we found that there is no available data for Am in poultry meat in our area. Few data sets are available for estimating the transfer of radionuclides in “soil-products” systems [8, 9] as the soil can lead to radioactive contamination of farm products [10, 11]. This paper describes the results of a field experiment with broilers on the transfer factor and concentration ratio in the “forage-organs” and “soil-organs” systems, the dynamics of accumulation and the equilibrium stage into different organs for ^241^Am and ^137^Cs. The review of the Russian language studies by Fesenko et al. [7] reports that the transfer of ^137^Cs, ^90^Sr, ^54^Mn, ^65^Zn to chicken tissue was described in four publications. The transfer factor F_*f*_ values for ^137^Cs varies from 0.7±0.1 (Mean±SD) to 2.3±0.3 in different tissues for a duration of 30–360 days. The generated concentration ratios (C_R_) of Am for the meat of different animals has been estimated as 1×10^−4^. Moreover, it is a fact that the migration of the radionuclide into meat or milk will significantly vary depending on the source of intake. For instance, forage contaminated by Cs has a higher bioavailability than ingested soil [12].

This study presents an investigation of the transfer parameters of ^137^Cs and ^241^Am to poultry products. The research focuses on the determination of the dynamics of the accumulation of radionuclides in different tissue of broilers and estimates transfer factor (F_*f*_) and concentration ratio (C_R_) values for broiler's meat after long-term feeding contaminated soil and forage.

## 2. Materials and methods

### 2.1. Study outline

The experiment was conducted at Institute of Radiation Safety and Ecology Experimental Farm [13], which is located in the STS territory. Objects of the study were 50±2 days old and 1700±100 g weight (live weight) broilers (cross *Arbor Acros*). They were purchased in a local poultry factory. All broilers were similar in clinical condition and external-constitutional data and they were kept in individual cages with a separate feeding and drinking bowl. During the experiment, the diet of all broilers was around 150–180 g/day/head (DW) commercial all-in-one granulated feed (hereinafter-all-in-one feed). Feeding was carried out twice a day in the morning (8.00–9.00 h) and evening (17.00–18.00 h). Before feeding, the all-in-one feed was steamed in warm water at a temperature of 20–25 °С until the water was completely absorbed and the granules swelled (12–20 minutes). For all broilers water was offered ad-libitum.

The broilers were divided into two groups, each group had nine subgroups and each subgroup had three broilers. In total 54 broilers were used. During the whole period of the experiment, all broilers were in the same conditions (housing systems, watering and feeding regime) except for the source of radionuclide intake into the body. Group 1 was fed with contaminated soil mixed with the all-in-one feed; the 2^nd^ group was fed with contaminated grass meal mixed with the all-in-one feed (see below). The duration of feeding with “contaminated” sources were 1, 2, 4, 8, 14, 28, 42, 56, 70 days (from each group one subgroup for each duration was used).

In vivo experiments were performed according to the guidelines of the ARRIVE and the European Communities Council Directive (86/609 EEC). All procedures were approved by the Special Community decision (protocol No. 40/20-06, Date 25.02.2014) of the Institute of Radiation Safety and Ecology NNC RK and the Veterinary Control and Oversight Committee of Kurchatov.

Group 1. Every morning each broiler was fed with a wet mixture of 30 g (DW) of soil and 50 g of all-in-one granulated feed. The broilers consumed the offered food in about 20–30 minutes. In the evenings, all-in-one granulated feed (100 g DW) was offered without any added soil.

Group 2. Every morning each broiler was fed with a wet mixture of 30 g (DW) of grass meal and 50 g of all-in-one granulated feed. The broilers consumed the offered food in about 30–40 minutes. In the evenings, all-in-one granulated feed (100 g DW) was offered without any added grass meal.

Soil for feeding was collected from a site of surface nuclear explosions test site called the "Experimental field" and which is known to have high levels of radioactive contamination. The test site territory fully described by Lukashenko [14]. Sampling was carried out at a distance of 20 meters from one of the funnel of a ground nuclear explosion. The upper 5 cm of soil was sampled from an area of 8–10 m^2^ and sieved through a 0.5-mm mesh. Sieved soil was placed in a container and mixed up thoroughly. Afterwards the soil was packaged in plastic bags of 30 g each. In total, about 700 bags of soil were prepared before the start of the experiment. The speciation of radionuclides in this soil has previously been determined by Kunduzbaeva [15] and sequential extraction data from this earlier study are presented in the Table 1.

**Table 1:** Form of radionuclides ^137^Cs and ^241^Am in the study soil [15].

Speciation of radionuclides is soil	^241^Am	^137^Cs
water-soluble(H2O)	˂1.3%	˂0.4%
exchangeable(1M CH3COONH4)	˂1.2%	˂0.7%
organic(0.1N NaOH)	˂1.6%	˂0.4%
mobile(1M HCl)	14.0%	˂0.6%
tightly bound	81.9%	97.9%

Study of radionuclide speciation in soils was conducted by sequential extraction as modified by Pavlotskaya [16]. The procedure was modified by adding an intermediate stage of determining fractions of organically bound radionuclides by 0.1 NaOH solution based on the technique developed by Tyurin [17]. The ratio of soil and leaching solution was 1:5.

Grass meal samples were made from vegetation taken from the test site territory called “Degelen”, which was designed for low-yield tests (up to several dozens of kilotons) in horizontal tunnels. At present, radionuclide contamination of the territory is caused due to the migration process of groundwater seepage through the epicenter [14]. At one of these sites, vegetation was mowed in mid of summer. The vegetation was presented meadow vegetation (*Chamaenerium angustifolium*, *Cirsium arvense*, *Tanacetum vulgare*, *Calamagrostis arundinacea*, *Urtica dioica*, *Veronica spuria*, *Mentha interrupta*, *Rumex confertus*, *Geranium collinum*, *Sanguisorba officinalis*, *Delphinium dictyocarpum etc*.). Mowed vegetation was washed to remove soil particles in the form of dust, dried to an air-dry state and ground to a state of grass meal (particle size 250 microns). Afterwards the grass meal was packaged in plastic bags of 30 g. In total, about 700 bags of grass meal were prepared before the experiment.

The daily intakes of radionuclides were estimated based upon activity concentrations (Bq kg^-1^, DW) in the all-in-one feed, soil and grass meal and the daily consumption (kg day^-1^ DW) of each of them. Observations of the feeding process confirmed that losses of the feed-soil and feed-grass meal feed mix due to spills or refused feed were minimal and comprised less than 5% of the total mixture volume. No faecal matter or refused feed was analyzed.

The research did not perform a blank experiment, except tree broilers’ samples (muscle, liver, and bone) which were analyzed at the beginning of the experiment. It was found that activity concentration of radionuclides ^137^Cs and ^241^Am in the samples were below detectable activity.

### 2.2. Sample preparation and radioanalysis

Samples of all-in-one feed, soil, grass meal and organs of all broilers (muscle, bone and liver) were taken for analysis. Three samples of all-in-one feed were taken before starting the feeding experiment from three different bags. Before Gamma-analysis, samples were ground by lab mill. Soil and grass meal samples were taken from the prepared feeding packages (5 packages each). Soil and grass meal samples preparation described above (see section 2.1).

The skin of the femoral muscle was removed, as well as the bone. Femoral bones from both legs were taken and cleaned from the muscle. At the beginning of experiment the fresh weight of the liver, bone, and muscle samples were 34±5 g, 49±5 g and 490±40 g, respectively. The fresh weight of the liver, bone, and muscle samples of broiler from ninth subgroup (70^th^ day of the experiment) achieved 41±8 g, 82±11 g and 1030±140 g, respectively. Organs were prepared for analysis by washing with flowing water, drying (to constant weight) followed by grinding/homogenizing using a lab mill. The dry weight of the liver, bone, and muscle samples was 8.7±0.8 g, 20±3 g and 140±14 g at the beginning and 11±2 g, 49±6 g and 260±40 g at the end of experiment. The dry weight of the liver, bone, and muscle samples was 8.9±1.4 g, 21±2 g and 120±10 g, respectively. All samples were accurately weighed into plastic containers for gamma analysis depending upon sample size.

Gamma-analyses were performed using a CANBERRA Ge BE3830 gamma radiation detector with a relative efficiency of 34% and Genie2000 software. For the energy calibration of spectrometers, a set of standard γ-sources (OSGI) was used; for geometry calibration, volumetric measures of specific activity (“OMACH” Rosatom) were used containing the following radionuclides: ^137^Cs, ^152^Eu, ^241^Am. The uncertainty is 40%. MDA for ^241^Am and ^137^Cs is 0.6 Bq kg^-1^. The methodology used for gamma-analysis has been described in the approved methods [18]. The laboratory has ISO 17025:2009 accreditation.

### 2.3. Estimation of transfer parameters

The standard parameter used to describe radionuclide input to farm animal organs is the transfer factor (F_*f*_; d kg^-1^) [5]:

$${\text{F}}_{f}=\frac{\text{Radionuclide}\;\text{activity}\;\text{concentration}\;\text{in}\;\text{animal}\;\text{organs}\left({\text{Bq}\;\text{kg}}^{-1}\;\text{fresh}\;\text{mass}\left(\text{FM}\right)\right)}{\begin{array}{l} \text{Daily}\;\text{intake}\;\text{of}\;\text{a}\;\text{radionuclide}\left({\text{Bq}\;\text{d}}^{-1}\right) \end{array}}$$

Following the recommendations of Beresford et al. [19] and the subsequent IAEA [5] publication, the dietary concentration ratio was also estimated where C_R diet_ is defined as:

$${\text{C}}_{\text{Rdiet}}=\frac{\text{Radionuclide}\;\text{activity}\;\text{concentration}\;\text{in}\;\text{animal}\;\text{organs}\left({\text{Bq}\;\text{kg}}^{-1}\;\text{FM}\right)}{\begin{array}{l} \text{Radionuclide}\;\text{activity}\;\text{concentration}\;\text{in}\;\text{the}\;\text{whole}\;\text{diet}\left({\text{Bq}\;\text{kg}}^{-1}\;\text{dry}\;\text{mass}\right) \end{array}}$$

## 3. Results & discussion

The live-weight of the broilers increased from 1700±100 g to 3300±200 g over the 70 days of the study; The mean (±SD) dry matter intakes of the study Group 1 and Group 2 broilers during the study was 180±10 g/day.

Activity concentrations of radionuclides measured in soil and grass meal samples are presented in Table 2. Each sources (soil, grass meal) contain kBq per kg of dry matter. The SD value of the mean activity concentration of radionuclides in soil and grass meal were not more that 10% and it is assumed that the activity concentration of radionuclides in the prepared sources was uniform in the total volume. As it was expected the activity concentrations of radionuclides (^137^Cs and ^241^Am) in the all-in-one feed was below the minimum detectable activity. Intakes of radionuclides by the study broilers, taking into account the mass of refused feed (not more than 5% of each source), are summarized in Table 3.

**Table 2:** Mean radionuclide activity concentration in soil and grass meal offered to the broilers during the experiment, Bq kg^-1^ DM.

Sample Number #	Soil (Group 1)		Sample Number #	Grass meal (Group 2)	
	^137^Cs	^241^Am		^137^Cs	^241^Am
1	1 600±100	340 000±30 000	1	5 800±300	620±30
2	1 400±100	430 000±40 000	2	5 700±300	550±30
3	1 600±100	420 000±40 000	3	5 500±300	570±30
4	1 500±100	440 000±40 000	4	5 500±300	640±30
5	1 500±100	430 000±40 000	5	5 700±300	660±30
Mean±SD	1 500±200	410 000±60 000	Mean±SD	5 600±300	610±60

**Table 3:** Radionuclide activity concentration in the whole diet and daily intake of radionuclides.

Group	Radionuclides (Mean±SD)	
	^137^Cs	^241^Am
Daily intake of radionuclides ^a^, Bq day^-1^ DM		
1^st^ group	46±8	12 000±1 500
2^nd^ group	170±30	18±3
Radionuclide activity concentration in the whole diet, kBq kg^-1^ DM		
1^st^ group	250±50	66 700±12 000
2^nd^ group	940±200	5 300±500

^a^ The SD associated with the daily activity reflective of differences in dry matter intake between broilers and SD of mean activity concentration of radionuclides in soil and grass meal

**Fig 1:**
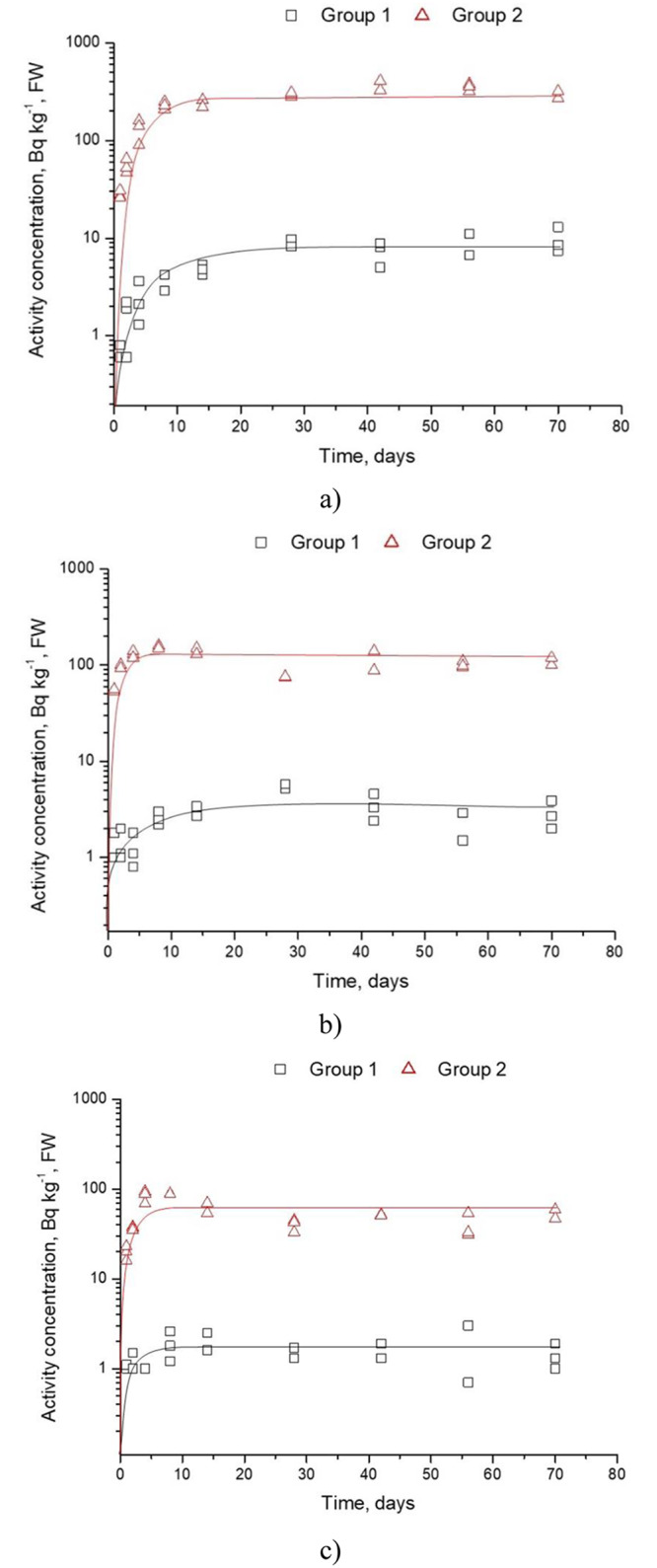
Mean (±SD) activity concentrations of ^137^Cs in the muscle (a), liver (b) and bone (c) during long term intake, Bq kg^-1^, FW (The analytical error of measurements no more than 10%).

**Fig 2:**
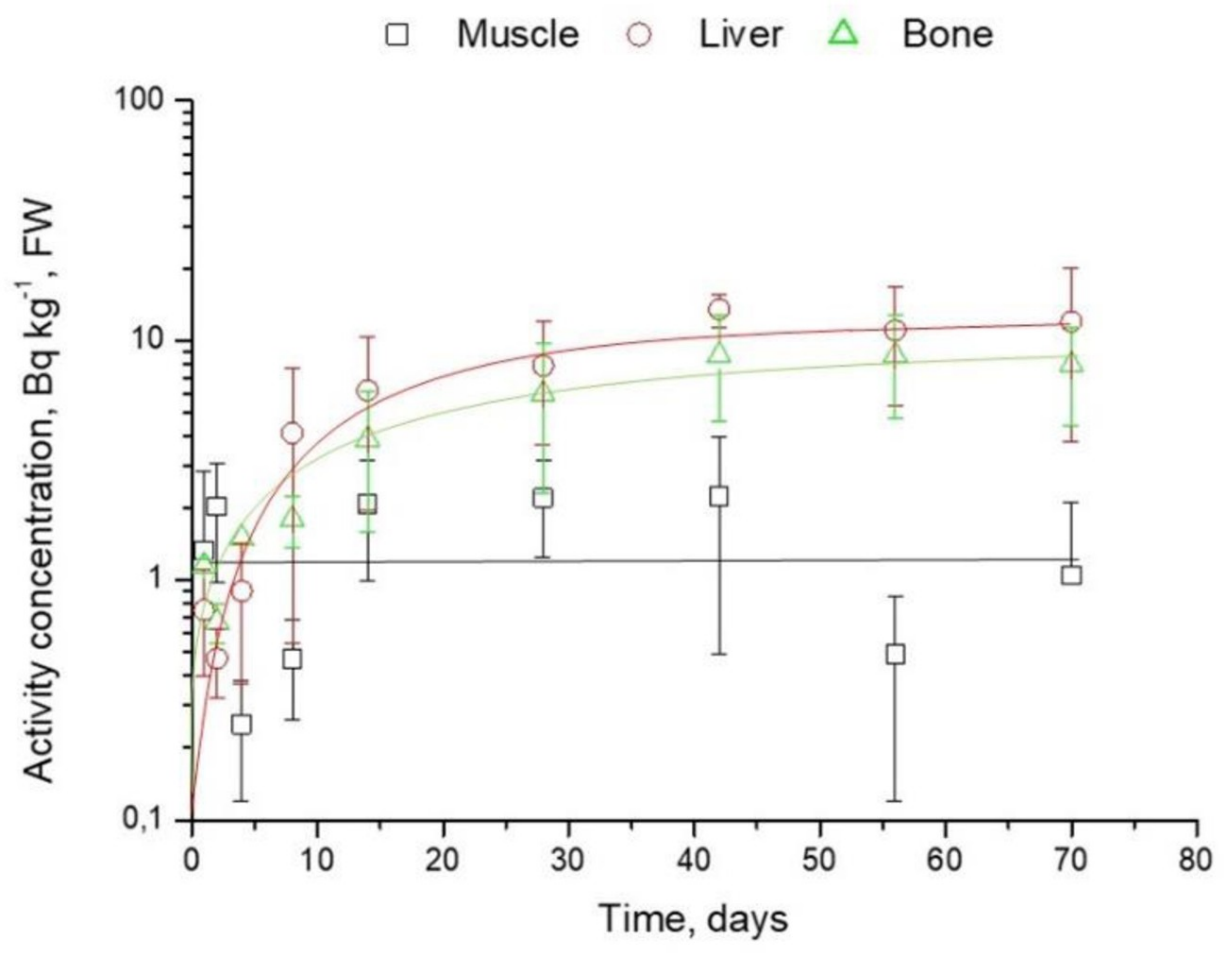
Mean (±SD) activity concentrations of ^241^Am in the organs of broilers Group 1 (n = 27) during long term intake, Bq kg^-1^,FW.

Figs 1 and 2 present the fresh weight (FW) radionuclide mean (±SD) activity concentration of ^137^Cs in the muscle, liver and bone of Group 1 and 2. ^137^Cs was detectable in almost all analyzed samples. Activity concentration of ^137^Cs in the muscle of broilers in Group 1 and 2 in last day of experiment reached 6–13 Bq kg^-1^ FW and 300–400 Bq kg^-1^ FW, respectively. At the same, time the activity concentration of ^137^Cs in the muscle is higher 2–3 and 5–6 times than in liver and bone, respectively. The relative activity concentrations in the different tissues of the broilers were generally as would be expected from the literature [20–22]

Unfortunately, the activity concentrations of ^241^Am in Group 2 were below the minimum detectable activity, but in Group 1 ^241^Am was detectable in almost all analyzed samples (Fig 2); as expected ^241^Am mainly deposited in liver and bone, muscle activity concentration is much lower than in others. Comparatively high concentrations in liver and bone are in agreement with previous observations on mammal species [19, 21]

### 3.1. Dynamics of accumulation

Fig 1 has shown the activity concentration of ^137^Cs in organs increases to a certain value, after which accumulation of radionuclides slow down, i.e. dynamic equilibrium occurs between accumulation and elimination. For bone and liver, it has come after 14 days, for muscle after 30 days. These data are in good agreement with the findings of previous studies [23] after ^137^Cs was administered as a chloride into the body. However, Sirotkin [24] showed that transfer factor of ^137^Cs of chicken meat for 30 days feeding was 2.4 times lower than with 390 days feeding. Astasheva [25] reported that biological half-life accumulation of adult geese muscles was 5 days, and for liver it was 1 day.

Compared to ^137^Cs, the deposition of ^241^Am in organs is different. Activity concentrations of ^241^Am in muscle are almost the same in the first and last day of the experiment, while in liver and bone it increased 5–10 times. Previously, it was determined that Am in farm [26, 27] and laboratory animals [28–30] accumulates in the liver and skeleton. The dynamics of transuranic elements’ metabolism in the liver can be explained by the dynamics of the exchange of complex compounds of the radionuclide with globulins. The figure (Fig 2) above shows that, similarly to ^137^Cs in muscle, ^241^Am in the bone swiftly increases in the first 40 days of the experiment, after that the growth of radionuclide activity slows down. However, in case of ^241^Am in the liver, it is not clear if it is exactly at the equilibrium stage, but it is obviously slowing down. In addition, it should be mentioned that the range of ^241^Am activity concentrations in organs in the same subgroups (n = 3) could be two times different, while for instance the range of ^137^Cs is less than 20%.

### 3.2. Transfer factor (F_*f*_) and concentration ratio (C_R_)

F_*f*_ and C_R_ were calculated for the equilibrium stage of radionuclides into the organs. For ^137^Cs in muscle equilibrium stage was considered after a duration of feeding more than 28 days (4 subgroups in each group), for liver and bone more than 14 days (5 subgroups in each group); for ^241^Am in liver and bone-more than 42 days (3 subgroups), for muscle-from first day of intake (all 9 subgroups). Table 4 shows that difference between Group 1 and 2 of F_*f*_ of ^137^Cs in broilers organs could reach one orders of magnitude while for C_R_ it reaches three orders of magnitude. F_*f*_ and C_R_ values to muscle tissue are higher than they are to the liver, regardless of the source of intake, by 2.5–3.5 times.

**Table 4:** Transfer factor and concentration ratio of ^137^Cs in broilers’ organs, mean±SD, 10^−2^.

Organs	N	Transfer factor			Concentration ratio		
		Group 1	Group 2	IAEA [5]	Group 1	Group 2	IAEA [5]
Muscle	11	18.8±4.6	192.9±25.8	270.0* (120–560)	0.073±0.018	34.7±4.7	39.0**
Liver	14	7.8±3.1	57.6±12.4	-	0.031±0.012	10.4±2.2	-
Bone	14	3.4±1.3	26.4±5.7	-	0.013±0.0052	4.7±1.0	-

*-Mean and range of F_*f*_ values for Cs in poultry meat which summarized 13 data including data for duck older than 40 days and duration of feeding with contaminated fed, not less than 20 days’;

**-generated values of C_R_ for all animals meat (beef, sheep and pork)

Comparing with summarized F_*f*_ values in the IAEA handbook [5] the obtained data for Group 2 is lower than the mean data, at the same time it is within the range of the data presented in the handbook. C_R_ for meat of broilers Group 2 (Table 4) is comparable with values generated for the meat of different animals [5]. It was found that F_*f*_ and C_R_ data that obtained for Group 1 is lower one and two orders of magnitude respectively, than it is other experiments where soil was used as a source of intake [8].

F_*f*_ of ^241^Am into broiler meat is lower by three orders of magnitude than it is for ^137^Cs. As the range of ^241^Am in muscle in equilibrium stage was high, it is recommended to use median data (Table 5). Our data review [1, 5, 6, 20] showed that transfer data for ^241^Am into chicken meat was not available yet. In comparison, the generated C_R_ values of ^241^Am for meat of different animals [5] is one order of magnitude higher than the obtained C_R_ data for Group 1. At the same time these differences for ^137^Cs could reach three orders of magnitude (Table 4).

**Table 5:** Transfer factor and concentration ratio of ^241^Am in broilers of group 1, ×10^−4^.

Organs	N	Transfer factor		Concentration ratio		
		Mean±SD	Median (Q_1/2_ –Q_3/4_)	Mean±SD	Median (Q_1/2_ –Q_3/4_)	IAEA [5]
Muscle	27	1.1±0.95	0.75 (1.4–0.33)	0.20±0.18	0.14 (0.28–0.06)	1.1*
Liver	6	10.1±4.5	10.0 (12.5–7.04)	1.8±0.82	1.8 (2.3–1.3)	-
Bone	9	7.03±2.8	9.2 (9.2–3.4)	1.3±0.50	1.7 (1.7–0.62)	

*-generated values of C_R_ for all animals’ meat

## 4. Conclusion

This paper presents transfer parameter data for ^241^Am, ^137^Cs to broilers’ meat, which is an important agricultural product for the inhabitants living nearby the STS. The study diets involved the feeding of contaminated soil and vegetation collected from within the STS. The transfer of radionuclides from ingested soil is generally lower, by up to one order of magnitude, than that from a diet including contaminated grass meal. The ingestion of soil and grass are a relevant source of radionuclide intake for feeding broilers on contaminated territory [11]. Our study has provided data for a poorly studied poultry meat, and in case of ^241^Am, a relatively poorly studied radionuclide with respect to transfer to animal products.

## Supporting information

S1 Appendix (DOCX)
