# Supplementary material for: The transfer of 241Am and 137Cs to the tissues of broilers’ organs
Source: PLoS One. 2020 Jul 1;15(7):e0235109. doi: 10.1371/journal.pone.0235109 (PMC7329069; doi:10.1371/journal.pone.0235109)
Supplement: S1 Appendix — (DOCX) [file pone.0235109.s001.docx]

Appendix

Data of Figure 1. Activity concentrations of ^137^Cs in the broiler’s organ, Bq kg^-1^, FW

| Duration of feeding the "contaminated" source, days | Group 1 | | | Group 2 | | | |
| --- | --- | --- | --- | --- | --- | --- | --- |
|  | muscle | liver | bone | muscle | liver | bone | |
| 1 | 0.6±0.1 | 1.8±0.9 | 1.0±0.6 | 27±5 | 54±11 | 16±3 | |
|  | 0.8±0.2 | BDA | 1.1±0.2 | 26±5 | 53±11 | 20±4 | |
|  | n.a. | n.a. | n.a. | 31±6 | 56±11 | 23±5 | |
| 2 | 1.9±0.4 | 1.1±0.2 | 1.5±0.6 | 47±9 | 100±20 | 38±8 | |
|  | 2.2±0.4 | 2.0±0.4 | BDA | 53±11 | 100±20 | 37±7 | |
|  | 0.6±0.1 | 1.0±0.2 | BDA | 65±13 | 93±19 | 35±7 | |
| 4 | 2.1±0.4 | 1.8±0.4 | 1.0±0.6 | 160±30 | 130±30 | 94±19 | |
|  | 3.6±0.7 | 0.8±0.2 | 0.1±0.02 | 140±30 | 140±30 | 89±18 | |
|  | 1.3±0.3 | 1.1±0.2 | BDA | 90±18 | 120±20 | 69±14 | |
| 8 | 2.9±0.6 | 2.2±0.4 | 1.8±0.8 | 250±50 | 160±30 | 89±18 | |
|  | 2.9±0.6 | 2.5±0.5 | 2.6±0.5 | 210±40 | 150±30 | 88±18 | |
|  | 4.2±0.8 | 3.0±0.6 | 1.2±0.6 | 230±50 | n.a. | n.a. | |
| 14 | 4.2±0.8 | 3.0±0.6 | 2.5±0.6 | 260±50 | 210±40 | 70±14 | |
|  | 5.3±1.1 | 3.4±0.7 | 1.6±0.4 | 220±40 | 130±30 | 54±11 | |
|  | 4.8±1.0 | 2.7±0.5 | 1.6±0.7 | n.a. | n.a. | n.a. | |
| 28 | 8.5±1.7 | 5.2±1.0 | 1.7±0.8 | 280±60 | 75±15 | 45±9 | |
|  | 9.7±1.9 | 5.2±1.0 | 1.7±0.5 | 300±60 | 76±15 | 33±7 | |
|  | 8.2±1.6 | 5.8±1.2 | 1.3±0.5 | 310±60 | 76±15 | 43±9 | |
| 42 | 5.0±1.0 | 2.4±0.5 | 1.9±0.8 | 330±70 | 88±18 | 52±10 | |
|  | 8.1±1.6 | 4.6±0.9 | 1.3±0.5 | 410±80 | 140±30 | 51±10 | |
|  | 8.8±1.8 | 3.3±0.7 | 1.3±0.5 | n.a. | n.a. | n.a. | |
| 56 | 6.7±1.3 | 2.9±0.6 | BDA | 380±80 | 110±20 | 54±11 | |
|  | 11.0±2.0 | 1.5±0.3 | 3.0±1.1 | 320±60 | 96±19 | 31±6 | |
|  | n.a. | n.a. | n.a. | 360±70 | 98±20 | 33±7 | |
| 70 | 13.0±3.0 | 3.9±0.8 | 1.9±0.5 | 270±60 | 100±20 | 59±12 | |
|  | 7.4±1.5 | 2.0±0.4 | BDA | 320±60 | 120±20 | 47±9 | |
|  | 8.5±1.7 | 2.7±0.5 | 1.3±0.4 | n.a. | n.a. | n.a. | |
| Note: BDA – below detectable activity, n.a.- not available. | | | | | | |  |

Data of Figure 2. Activity concentrations of ^241^Am in the broiler’s organ, Bq kg^-1^, FW

| Duration of feeding the "contaminated" source, days | Group 1 | | | Group 2 | | |
| --- | --- | --- | --- | --- | --- | --- |
|  | muscle | liver | bone | muscle | liver | bone |
| 1 | 0.4±0.1 | 1±0.2 | 1.2±0.3 | BDA | BDA | BDA |
|  | 0.5±0.1 | BDA | 1.1±0.4 | BDA | BDA | BDA |
|  | n.a. | n.a. | n.a. | BDA | BDA | BDA |
| 2 | 3.1±0.6 | 0.5±0.1 | 0.8±0.3 | 0.4±0.1 | BDA | BDA |
|  | 1.0±0.2 | 0.3±0.1 | BDA | BDA | BDA | BDA |
|  | 2.0±0.4 | 0.6±0.1 | BDA | BDA | BDA | BDA |
| 4 | 0.4±0.1 | 1.3±0.3 | 1.5±0.3 | 0.5±0.1 | BDA | BDA |
|  | 0.15±0.03 | 0.3±0.1 | BDA | BDA | BDA | BDA |
|  | 0.19±0.03 | 1.1±0.2 | BDA | 0.4±0.1 | BDA | BDA |
| 8 | 0.4±0.1 | 2.0±0.4 | 1.3±0.3 | 1.0±0.2 | BDA | BDA |
|  | 0.7±0.1 | 2.1±0.4 | 2.1±0.4 | BDA | BDA | BDA |
|  | 0.3±0.1 | 8.2±1.6 | 2.0±0.4 | BDA | BDA | BDA |
| 14 | 1.6±0.3 | 11±2 | 6.5±1.3 | BDA | BDA | BDA |
|  | 3.3±0.7 | 4.2±0.8 | 2.5±0.5 | BDA | BDA | BDA |
|  | 1.3±0.3 | 3.3±0.7 | 2.6±0.5 | n.a. | n.a. | n.a. |
| 28 | 3.3±0.7 | 3.7±0.7 | 2.7±0.5 | BDA | BDA | BDA |
|  | 1.6±0.3 | 7.8±1.6 | 5.3±1.1 | BDA | BDA | BDA |
|  | 1.7±0.3 | 12±2 | 10.0±2.0 | BDA | BDA | BDA |
| 42 | 4.2±0.8 | n.a. | 11.0±2.0 | BDA | BDA | BDA |
|  | 1.6±0.3 | 12±2 | 11.0±2.0 | BDA | BDA | BDA |
|  | 0.9±0.2 | 15±3 | 4.0±1.0 | n.a. | n.a. | n.a. |
| 56 | 0.4±0.1 | 15±3 | 11.0±2.0 | 0.3±0.1 | BDA | BDA |
|  | 0.9±0.2 | 7.0±1.4 | 11.0±2.0 | 0.3±0.1 | BDA | BDA |
|  | n.a. | n.a. | n.a. | BDA | BDA | BDA |
| 70 | 0.14±0.03 | 9.9±2.0 | 4.1±0.8 | BDA | BDA | BDA |
|  | 0.8±0.2 | 5.0±1.0 | 8.7±1.7 | BDA | BDA | BDA |
|  | 2.2±0.4 | 21±4 | 11.0±2.0 | n.a. | n.a. | n.a. |
| Note: BDA – below detectable activity, n.a.- not available. | | | | | | |
